# Grifolin inhibits tumor cells adhesion and migration via suppressing interplay between PGC1α and Fra-1/LSF-MMP2/CD44 axes

**DOI:** 10.18632/oncotarget.11929

**Published:** 2016-09-10

**Authors:** Xiangjian Luo, Namei Li, Juanfang Zhong, Zheqiong Tan, Ying Liu, Xin Dong, Can Cheng, Zhijie Xu, Hongde Li, Lifang Yang, Min Tang, Xinxian Weng, Wei Yi, Jikai Liu, Ya Cao

**Affiliations:** ^1^ Key Laboratory of Carcinogenesis and Invasion, Chinese Ministry of Education, Xiangya Hospital, Central South University, Changsha, Hunan 410078, China; ^2^ Cancer Research Institute, Xiangya School of Medicine, Central South University, Changsha, Hunan 410078, China; ^3^ Key Laboratory of Carcinogenesis, Chinese Ministry of Health, Changsha, Hunan 410078, China; ^4^ Department of Medicine, Hunan Traditional Chinese Medical College, Zhuzhou, Hunan 412000, China; ^5^ School of Pharmacy, South-Central University For Nationalities, Wuhan, Hubei 430074, China

**Keywords:** grifolin, PGC1α, ROS, adhesion, migration

## Abstract

Grifolin, a farnesyl phenolic compound isolated from the fresh fruiting bodies of the mushroom *Albatrellus confluens*, exhibits effective antitumor bioactivity in previous study of our group and other lab. In this study, we observed that grifolin inhibited tumor cells adhesion and migration. Moreover, grifolin reduced reactive oxygen species (ROS) production and caused cellular ATP depletion in high-metastatic tumor cells. PGC1α (Peroxisome proliferator-activated receptor γ, coactivator 1α) encodes a transcriptional co-activator involved in mitochondrial biogenesis and respiration and play a critical role in the maintenance of energy homeostasis. Interestingly, grifolin suppressed the mRNA as well as protein level of PGC1α. We further identified that MMP2 and CD44 expressions were PGC1α inducible. PGC1α can bind with metastatic-associated transcription factors: Fra-1 and LSF and the protein-protein interaction was attenuated by grifolin treatment. Overall, these findings suggest that grifolin decreased ROS generation and intracellular ATP to suppress tumor cell adhesion/migration via impeding the interplay between PGC1α and Fra-1 /LSF-MMP2/CD44 axes. Grifolin may develop as a promising lead compound for antitumor therapies by targeting energy metabolism regulator PGC1α signaling.

## INTRODUCTION

During the process of metastasis, two of the critical steps of metastasis are adhesion and migration of the primary tumor cell on the extracellular matrix (ECM) at the distant site. Cell-ECM interactions (i.e. cell attachment to, spreading on, and movement along the ECM) are essential for the tumor cells to adapt to the new metastatic site microenvironment [1–4]. The interactions between cancer cells and ECM are dependent upon cell adhesion molecules (CAMs), such as cadherins and lymphocyte homing receptors (CD44). CAMs are critical for cell adhesion [5, 6]. After attachment, metastatic cells perform penetration of the ECM by matrix cleaving to facilitate further migration and invasion. The major enzymes responsible for matrix degradation are matrix metalloproteases (MMPs) [4].

Mitochondrial oxidative stress leading to oxidative phosphorylation (OxPhos) stimulation and/or enhanced ROS production, can act as key drivers of the malignant changes in primary tumors to promote their progression to metastasis [7]. Cancer cells that have been shed from a primary tumor need to overcome the energy deficit to survive and form metastases. Consistent with the requirements, migratory/invasive cancer cells specifically favor mitochondrial respiration and increased adenosine triphosphate (ATP) production [7, 8]. ROS accumulation in hypoxic cancer cells induces expression of PGC-1α [7, 9, 10]. PGC1α encodes a transcriptional co-activator involved in energy homeostasis, fatty acids oxidation and glucose metabolism [11]. Mitochondrial biogenesis and respiration induced by PGC1α are essential for functional motility of cancer cells [12]. PGC1α powerfully regulated VEGF expression and angiogenesis in cultured muscle cells and skeletal muscle in vivo [13]. Clinical analysis of human invasive breast cancers revealed a strong correlation between PGC1α expression in invasive cancer cells and the formation of distant metastases [14]. Whereas specific inhibitors of PGC1α are not yet available, the major strategy to disrupt PGC1α signaling have been focused on blocking key enzymes of PGC1α-dependent metabolic pathways or targeting the interaction between PGC1α and its associated transcription factors [14–18].

Grifolin, a secondary metabolite isolated from the fresh fruiting bodies of the mushroom *Albatrellus confluens*, has shown various pharmacological and microbiological effects [19, 20]. Grifolin exhibited inhibitory activity against nitric oxide (NO) production in RAW 264.7 cells [21]. Grifolin and its derivatives presented novel partial agonists for free fatty acid receptors GPR120 [22]. The anticancer activities of grifolin were first reported by our group [23–27]. Recently we identified that grifolin can directly bind to extracellular regulated protein kinases 1/2 (ERK1/2) and inhibits ERK1/2 kinase activities [28]. Here, we demonstrated the anti-adhesion and migration effect of grifolin, its role in ROS production and cellular ATP depletion, and further investigated the interplay between PGC1α and Fra-1/LSF-MMP2/CD44 axes suppressed by grifolin.

## RESULTS

### Grifolin suppresses migration and adhesion of metastatic carcinoma cells

**Figure 1:**
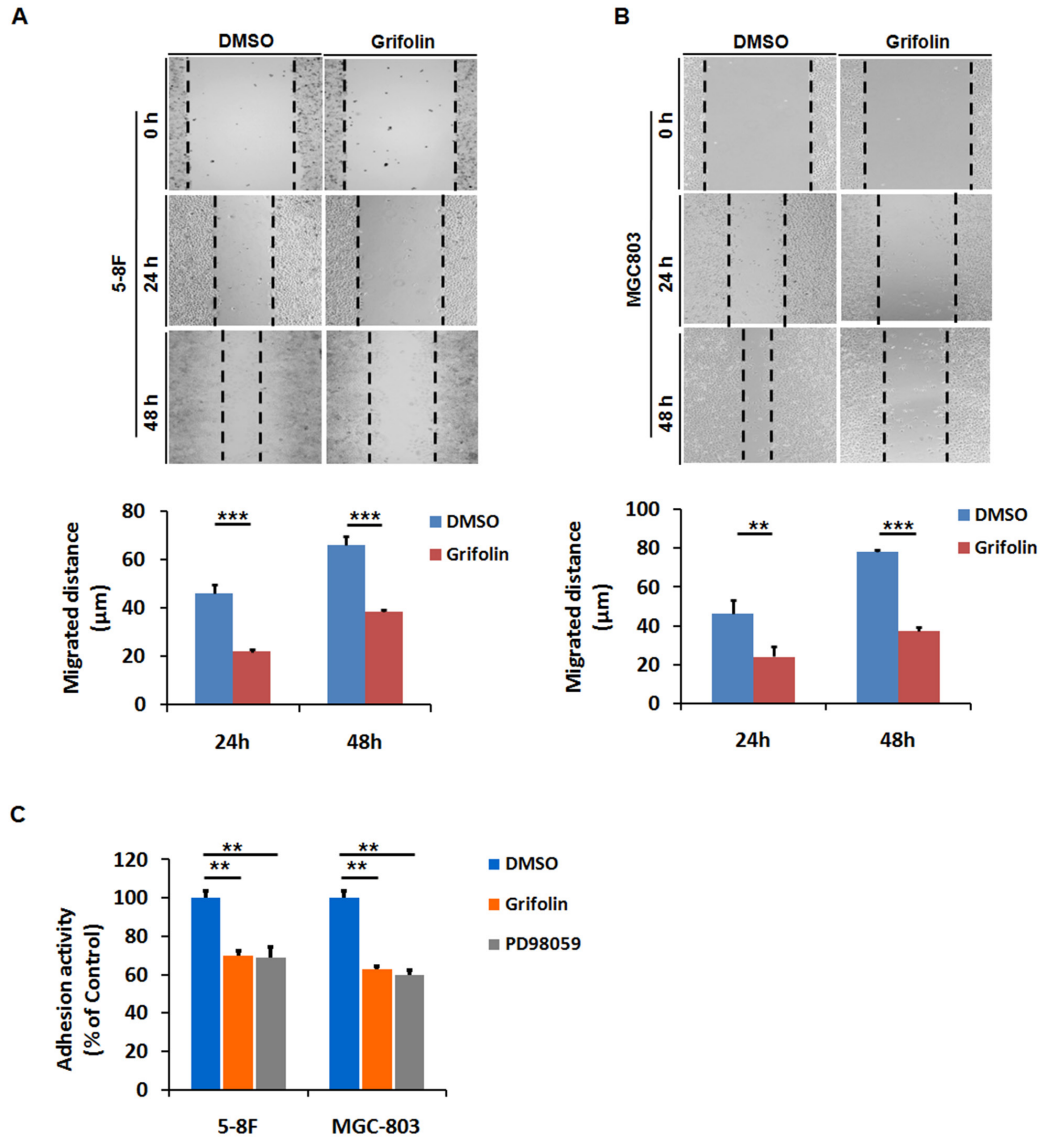
Grifolin inhibits migration and adhesion of high-metastatic cancer cells Grifolin suppresses migration in **A.** 5-8F, **B.** MGC803 cells using wound healing assay. Cells were scraped, and the migration ability of the cells in the presence of DMSO (control) or 10μM grifolin in low-serum condition was monitored with an inverted microscope. After 24 h or 48 h treatment, gap distances of the wounded regions in each group were measured from four independent experiments. **C.** Grifolin decreases adhesion of 5-8F and MGC-803 cells. Cells were pretreated with DMSO or grifolin for 24 hours prior to the adhesion assays, followed by being placed in wells coated with matrigel and allowed to adhere for 40 min. After removing the unattached cells by washing, the attached cells were quantified by measurement of cellular activity through MTS test. Absorbance at 490nm was measured using a microplate reader. Data are shown as mean values ± S.D. of independent, triplicate experiments. The asterisks (**, ***) indicate significant differences (*p* < 0.01, *p*<0.001, respectively) compared to the DMSO control.

Since cellular motility is an essential part of cancer metastasis, to identify the anti-migration effect of grifolin, we performed the wound healing assay in highly metastatic 5-8F, MGC803 cells. After 24 or 48 h treatment with 10μM grifolin in low-serum condition, the migratory capacity of 5-8F cells was significantly reduced compared to the DMSO control (Figure 1A). Likewise, inhibitory effects of grifolin were also observed in MGC803 (Figure 1B) and MDA-MB-231 cells (Supplementary Figure S1A).

Cell adhesion is interrelated with cellular motility and play a decisive role in metastatic spread [29]. We further examine the anti-adhesion effect of grifolin. After 24 h treatment with 40 μM grifolin, cells were added onto the matrigel-coated wells in serum-free medium and allowed to adhere for an additional 40 min before measurement. The number of adhensive 5-8F cells significantly decreased by 29.5% in the presence of grifolin compared to the DMSO control, so did in MEK1 inhibitor PD98059 treated group (Figure 1C). Similar effects of grifolin were further confirmed in MGC-803 (37.2%) (Figure 1C) and MDA-MB-231 cells (40.9%) (Supplementary Figure S1B) after 40μM grifolin treatment.

### Effects of Grifolin on ROS production and cellular ATP depletion

**Figure 2:**
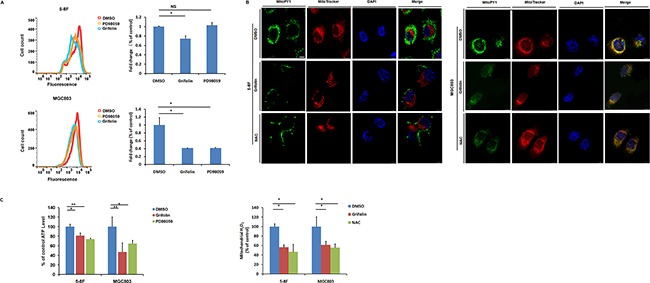
Grifolin attenuates intracellular ROS level and causes ATP depletion **A.** Intracellular superoxide level of cells after treatment with DMSO, grifolin (40μM) and PD98059 (40μM) were measured by dihydroethidine staining. **B.** Cells were treatment with DMSO, grifolin (40μM) or ROS inhibitor NAC (10mM) for 24 h. Mitochondrial ROS levels were determined by using a specific mitochondrial H_2_O_2_ probe, MitoPY1 and imaged by confocal laser microscopy. Quantitative determination of the fluorescent intensity of MitoPY1 (green) was performed as described under “Materials and Methods”. MitoTracker, a mitochondrion-selective probe. Scale bar, 10μm. **C.** Cells were treated with DMSO, grifolin (40μM) or PD98059 (40μM) for 24 h and ATP levels of each group were determined using a luminescent ATP assay. Data are shown as mean values ± S.D. of independent, triplicate experiments. The asterisks (*,**) indicate significant differences (p < 0.05, p < 0.01, respectively) compared to the DMSO control. NS, no significance.

Oxidative stress can promote tumorigenesis [30]. Cancer cells exhibit elevated ROS and ROS can accelerate cells invasive properties [31, 32]. To test this in our cellular context, intracellular ROS levels of 5-8F and MGC803 cells were analyzed. Cells were treated with DMSO or grifolin (40μM) for 24 h and then harvested and suspended in PBS. Superoxide was measured by dihydroethidine staining of the cells followed by flow cytometry analysis. Grifolin treatment of the cells resulted in marked decrease in fluorescence intensity indicating a greatly decreased superoxide level compared to that of control in 5-8F and MGC803 cells, respectively (Figure 2A). Furthermore, grifolin inhibited mitochondrial ROS levels by ∼40% compared to the DMSO control using a specific mitochondrial H_2_O_2_ probe, MitoPY1. Consistently, treatment with antioxidant, N-acetylcysteine (NAC) decreased ROS levels to that of control as well (Figure 2B). Since the tumor requires high levels of ATP for survival, proliferation and metastasis, we then investigated whether grifolin treatment impairs cellular ATP production in tumor cells. As shown in Figure 2C, the ATP level of the grifolin treated cells declined markedly compared to the control cells.

### Grifolin blockades CD44 and MMP2 expression and activity

**Figure 3:**
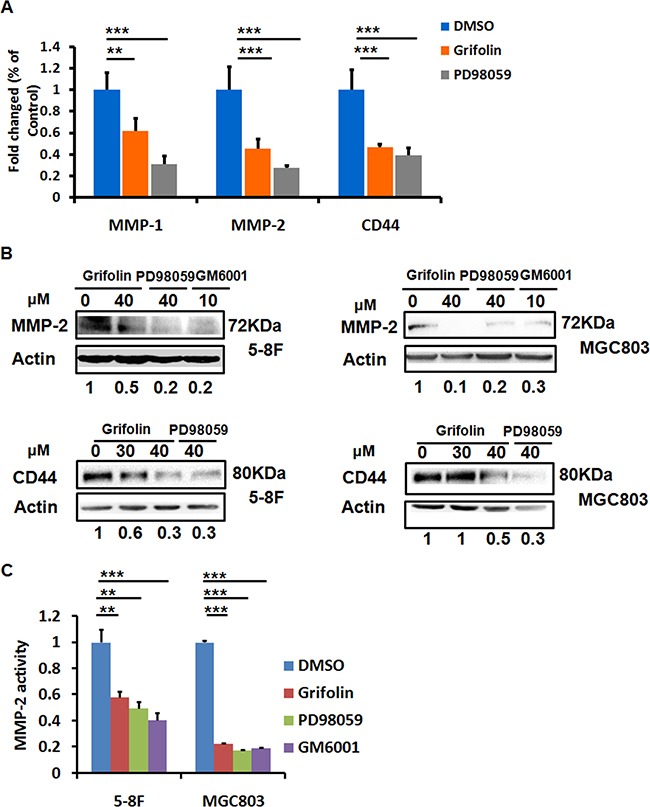
Grifolin suppresses MMPs and CD44 expressions **A.** Inhibition of MMPs and CD44 mRNA levels by grifolin. 5-8F cells were treated with DMSO, grifolin (40μM) or PD98059 (40μM) for 24 h. The mRNA expression of MMPs and CD44 were examined by real-time PCR, and GAPDH was served as the normalization control. **B.** Blockade of MMP2 and CD44 protein expressions by grifolin. 5-8F and MGC803 Cells were cultured and treated as designated in the figure for 24 h, then MMP2 and CD44 expressions were detected by western blot. Actin served as a loading control. **C.** Effect of grifolin on the enzymatic activity of secreted MMP-2. 5-8F and MGC803 cells were treated with DMSO, grifolin(40μM), PD98059(40μM) or GM6001(10μM), respectively for 24h. The amount of secreted MMP-2 activity were determined by the SensoLyte® 520 MMP-2 assay and the fluorescence was monitored at excitation/emission wavelengths of 490 nm/520nm. Data are shown as mean values ± S.D. of independent, triplicate experiments. The asterisks (**,***) indicate significant differences (*p* < 0.01, *p* < 0.001, respectively) compared to the DMSO control.

The process of cell invasion is a combination of cell migration with concurrent degradation of the surrounding ECM. This degradation of ECM proteins is mediated largely by matrix metalloproteinases (MMPs). MMP-2, along with MMP-9, makes up the gelatinase family of MMPs. To explore the mechanism behind the antitumor activity of grifolin, we performed a screening of genes related to tumor adhesion/invasion regulation, including MMP1,2,3,9,19, TIMP-1,2,3, CD44, CDH1 and PCDH10. The data showed that after grifolin treatment the most markedly decreased genes are MMP2 and CD44 (Figure 3A, Supplementary Figure S2). Inhibition of MMP2 and CD44 expressions were further confirmed at the protein level by western blot in 5-8F and MGC803 cells (Figure 3B). Impeding of the ERK1/2 pathway by use of PD98059 abated the expression of MMP2 and CD44 as well. GM6001, a MMPs inhibitor, served here as a positive control.

The metastatic tumor cells produce secreted MMP-2. To evaluate the activity of secreted MMP-2 modulated by grifolin, a MMP2 enzymatic assay was conducted by using a 5-FAM/QXL™520 fluorescence resonance energy transfer (FRET)^6^ peptide as a substrate. In the intact FRET peptide, the fluorescence of 5-FAM is quenched by QXL™520. Upon cleavage into two separate fragments by MMP-2, the fluorescence of 5-FAM is recovered, and can be monitored at excitation/emission wavelengths of 490 nm/520 nm. 5-8F and MGC803 cell media conditioned in the presence of DMSO, grifolin, PD98059 or GM6001, respectively for 24 h were collected. We then isolated MMP-2 from the cell culture media by immuno-affinity purification using an antibody specific against MMP-2. After adding MMP-2 substrate to the enzymatic reaction system, fluorescence detection were performed. The results showed a significant enzymatic activity suppression in grifolin treated group, the similar effects were also found in cells treated with PD98059 or GM6001. As shown in Figure 3C, the secreted MMP-2 decreased significantly with the inhibition rate of 41.8% and 77.2% in 5-8F and MGC803 cells, respectively. Thus, the data suggest that grifolin effectively suppresses the enzymatic activity of MMP-2 in high metastatic tumor cells and the action is associated with ERK1/2 signaling.

Adhesion molecule CD44 binds to several components of the ECM, such as fibronectin, HA and laminin, to take part in cell filopodia formation and associate with cell migration and invasion [33]. In our previous study, we observed that grifolin effectively suppressed filopodia formation in high metastatic 5-8F and MGC803 cells, which also support our proposal that grifolin inhibits cell aggressive phenotype by blockade of MMPs and CD44 expression. Taken together, our present findings implicate that the blockade of MMP2 and CD44 expressions as well as MMP-2 activity may contribute to the inhibitory effect of grifolin on tumor cell migration and adhesion.

### Inhibition of PGC1α by grifolin contributes to its anti-migration and adhesion effect

**Figure 4:**
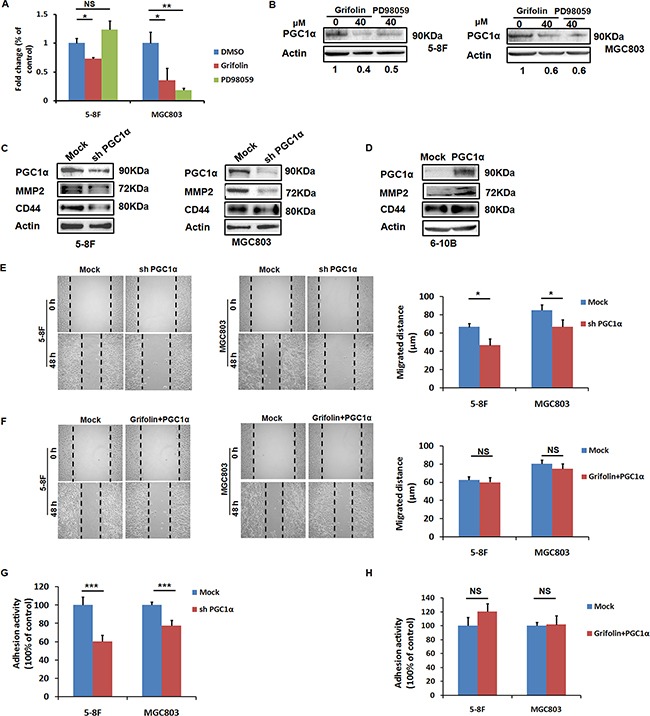
PGC1α induces MMP2 and CD44 expressions and is involved in the anti-migration and adhesion effect of grifolin **A.** Grifolin declines PGC1α mRNA levels. 5–8F and MGC803 cells were treated with DMSO, grifolin (40μM) or PD98059 (40μM) for 24 h. Total RNA was isolated from cells and subjected to real-time PCR. **B.** Grifolin inhibits PGC1α protein expression. 5–8F and MGC803 cells were treated with DMSO, grifolin (40μM) or PD98059 (40μM) for 24 h. Cell lysates were prepared and examined by western blot. Actin served as a loading control. **C.** Downregulation of MMP2 and CD44 expressions as a result of PGC1α inhibition by shRNA. 5–8F and MGC803 cells were transfected with PGC1α shRNA (shPGC1α) or control shRNA (Mock) for 72 h, then PGC1α, MMP2 and CD44 expressions were examined by western blot analysis. **D.** MMP2 and CD44 are upregulated as the result of ectopic expression of PGC1α. 6-10B cells were transfected with PGC1α expression vector p GV287- PGC1α or mock vector for 48 h, then PGC1α, MMP2 and CD44 expressions were detected by western blot analysis. **E.** Depletion of PGC1α attenuates migratory capacity of tumor cells. 5-8F and MGC803 cells were transfected with PGC1α shRNA or control shRNA for 72 h, respectively, and the migratory capacity of cells was examined using wound-healing assay. **F.** Overexpression of PGC1α reverses the anti-migratory effect of grifolin. 5-8F and MGC803 cells were treated with grifolin for 24 h followed by ectopic PGC1α expression, then the migratory capacity of cells was examined using wound-healing assay. **G.** Depletion of PGC1α attenuates adhesive capacity of tumor cells. 5-8F and MGC803 cells were transfected with PGC1α shRNA or control shRNAfor 72 h, respectively, then the adhesive capacity of cells was examined using adhesion assay. **H.** Overexpression of PGC1α reverses the anti-adhesive effect of grifolin. 5-8F and MGC803 cells were treated with grifolin for 24 h followed by ectopic PGC1α expression, then the adhesive capacity of cells was examined. Data are shown as mean values ± S.D. of independent, triplicate experiments. The asterisks (*,**,***) indicate significant differences (*p* < 0.05, *p* < 0.01, *p* < 0.001, respectively) compared to the DMSO control. NS, no significance.

ROS (H_2_O_2_) can induce PGC1α expression in cancer cells and in turn drive expression of a series of genes involved in oxidative metabolism, many of which overlap with those pro-metastatic genes regulated by the hypoxia-inducible factor (HIF) transcription factors, such as VEGF [7]. As we have illustrated that grifolin dampened ROS production in high-metastatic tumor cells, it prompted us to further examine the effect of grifolin on PGC1 expression. We demonstrated that treatment with grifolin attenuated the mRNA level of PGC1α compared to the DMSO control (Figure 4A). Inhibition of PGC1α expression was further confirmed at the protein level (Figure 4B).

We wondered whether there exists interplay between PGC1α and MMP2/CD44 signaling, we transfected 5-8F and MGC803 cells with mock or PGC1α shRNA for 72 h. Western blot showed successful PGC1α depletion following transfection, whilst mock shRNA did not affect PGC1α expression. In the meanwhile, the depletion of PGC1α significantly decreased MMP2 and CD44 (Figure 4C). Conversely, overexpression of PGC1α induced MMP2 and CD44 expressions in non-metastatic nasopharyngeal carcinoma 6-10B cells (Figure 4D). Therefore, we confirmed that MMP2 and CD44 expressions were PGC1α inducible. To further ascertain whether PGC1α mediates the observed effects of grifolin, PGC1α shRNA was transfected to 5-8F and MGC803 cells, respectively. The depletion of PGC1α suppressed migratory and adhesive capacities of tumor cells (Figure 4E, 4G), as did by grifolin treatment (Figure 1). In the recovery experiment, 5-8F and MGC803 cells were treated with grifolin followed by ectopic PGC1α expression. This rescued the aggressive phenotype of tumor cells suppressed by grifolin (Figure 4F, 4H). Altogether, these results show that the anti-migration and adhesion effect of grifolin, if not all, at least partially depends on inhibition of PGC1α.

### Grifolin interferes the interplay between PGC1α and Fra-1/LSF-MMP2/CD44 axes

**Figure 5:**
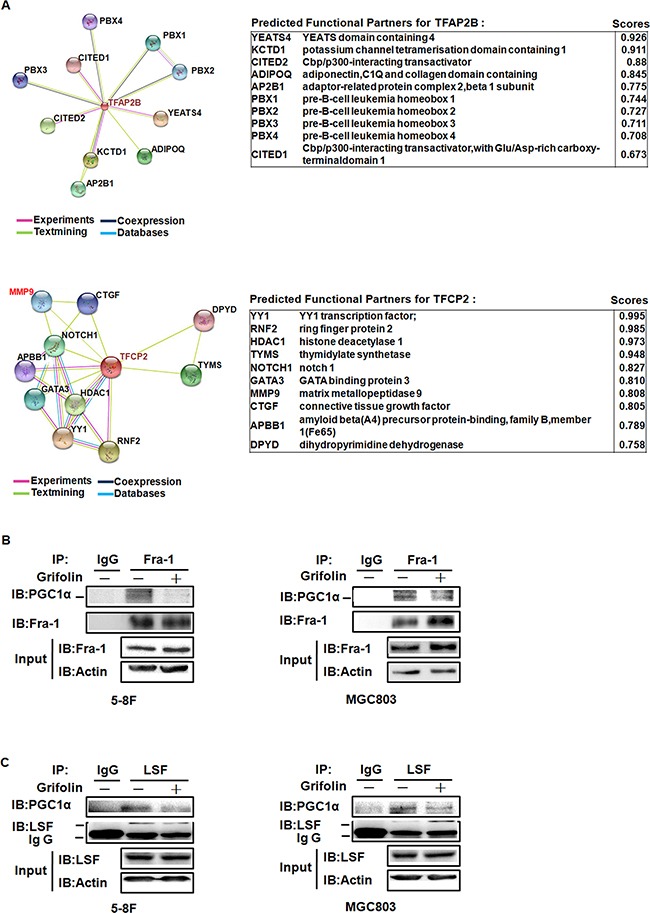
The illustration of TFs that binding to PGC1α and PPI interfered by grifolin treatment **A.** This is the evidence view of the functional partners to the central protein by PPI prediction. Different line colors represent the types of evidence for the association. Left, TFAP2B (coding for AP-2β). Right, TFCP2 (coding for LSF). **B.** 5-8F and MGC803 cells were treated with grifolin (40μM) for 24 h and cell lysates were immunoprecipitated with anti-Fra-1 antibody and analyzed by western blot for PGC1α. **C.** 5-8F and MGC803 cells were treated with grifolin (40μM) for 24 h and cell lysates were immunoprecipitated with anti-LSF antibody and analyzed by western blot for PGC1α. Immunoprecipitation using non-immune IgG was used in parallel as a control.

The PGC1α coactivators have powerful transcriptional activity when linked to a heterologous DNA binding domain or when they dock on a transcription factor [34]. To further seek for the specific transcription factor (TF) binding to PGC1α, we performed a Co-IP pulldown assay using anti-PGC1α antibody followed by LC/MS/MS. Among 300 peptides identified, only two TFs, AP2βand LSF were found bound to PGC1α-Protein G agarose complex (Supplementary Table S2). Moreover, using STRING - Protein-Protein Interactions database prediction, there is functional relation between LSF (also known as TFCP2) and MMPs (Figure 5A). Transcription factor binding-site prediction analysis using SABiosciences Text Mining Application and the UCSC Genome Browser further suggested that there exist binding sites of activating protein-1(AP-1) family members in the promoter regions of CD44 and MMP2.

**Figure 6:**
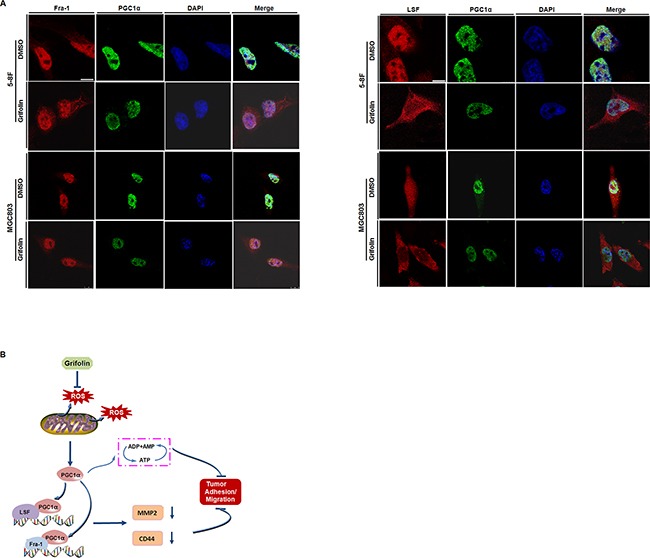
Mechanical schematic illustration of impediment of tumor cells migration and adhesion by grifolin **A.** Immunofluorescence microscopy analysis of the co-localization of PGC1α with Fra-1 or LSF. 5-8F and MGC803 cells were treated with DMSO or grifolin (40μM) for 24 h. PGC1α was stained with anti-PGC1α antibody, followed by Alexa-Fluor-488- conjugated anti-mouse-IgG secondary antibody. And Fra-1 or LSF was labeled with corresponding antibody respectively, followed by Alexa-Fluor-594-conjugated anti-rabbit-IgG secondary antibody. The nuclei were stained with DAPI. Scale bar, 10μm. **B.** Grifolin inhibits ROS production in cancer cells to decrease PGC1α expression. The downregulation of PGC1α induces ATP depletion. PGC1α binds to metastatic-associated transcription factor LSF and/or Fra-1 to promote MMP2 and CD44 expressions. Grifolin suppresses the interplay between PGC1α and MMP2/CD44 signaling, which contributing to the impediment of tumor cell migration/adhesion.

Accordingly, using co-Immunoprecipitation assay, we examined whether there exists protein-protein interaction (PPI) between PGC1α and TFs, AP-1 (Fra-1/c-Jun) and LSF and how the PPI interfered by grifolin. Endogenous PGC1α was co-immunoprecipitated with Fra-1 or LSF after grifolin treatment in 5-8F and MGC803 cells, respectively. It showed that PGC1α could be detected by Western blotting of Fra-1 and LSF immunoprecipitates. Compared to the DMSO control, the interaction of PGC1α/Fra-1 or PGC1α/LSF abated markedly with grifolin treatment (Figure 5B–5C). While we were not able to immunoprecipitate PGC1α with the c-Jun complex (Supplementary Figure S3). Immunofluorescence staining also revealed that grifolin attenuated the nucleic co-localization of PGC1α with Fra-1 or LSF in 5-8F and MGC803 cells, respectively (Figure 6A). Collectively, all the data indicate that grifolin may interrupt PPIs of PGC1α/Fra-1 and PGC1α/LSF to inhibit MMP2 and CD44 expressions.

## DISCUSSION

Considerable attention has focused on the cancer chemopreventive and therapeutic effect of natural compounds, which originate mainly from botany, fungi and marine organism [27, 35]. Our study demonstrated that grifolin, a natural farnesyl polyphenol chemical from the mushroom *Albatrellus confluens*, blocked ROS production and induced ATP depletion to inhibit tumor cells adhesion/migration. The mechanism was due to the suppression of PGC1α expression as well as the interplay between PGC1α and Fra-1/LSF-MMP2/CD44 signaling interrupted by grifolin.

Evidence is accumulating that changes in the ability of cancer cells to adhere to ECM play a decisive role in metastatic spread. The present study demonstrated that grifolin exerted dramatical inhibitory effect on the cell adhesion and migration in high metastatic tumor cells. Gene expression analysis revealed a marked decrease in MMP-2 and CD44 (P<0.001), a modest reduction in MMP-1(P<0.01) with grifolin treatment. However, we did not observe differences in MMP-3,9,19 expressions. It may be partially owing to low endogenous mRNA level of MMP-9 and MMP-19 in 5-8F cells. Inhibition of MMP2 and CD44 expressions by grifolin were further confirmed at the protein level in 5-8F and MGC803 cells. Moreover, we confirmed the suppression of MMP2 enzymatic activity with grifolin treatment. It was also consistent with our previous study that grifolin epigenetically upregulated TIMP-1,2 gene transcription [28]. Therefore, it indicates that grifolin may impede metastatic tumor cells adhesion and migration through inhibition of CD44 and MMP2 expression and function as well. Noteworthy, CD44 acts as stem cell surface marker in both nasopharyngeal carcinoma and gastric cancer [36, 37], the inhibition of CD44 expression by grifolin may contribute to its inhibitory effect on tumor invasion.

OxPhos stimulation and/or Enhanced ROS production are essential for promoting and sustaining the highly metastatic phenotype in tumor cells [7]. It is worthy to note that PGC-1α is a positive regulator of mitochondrial biogenesis and respiration and play a critical role in the maintenance of energy homeostasis [11]. Here we reported that grifolin suppressed intracellular ROS production and notably inhibited the mitochondrial ROS levels to that of control cells, then induced cellular ATP depletion in metastatic cancer cells. LeBleu, V.S. et al. described that quantitative PCR analyses showed specific upregulation of PGC-1α in circulating tumour cells compared with primary tumours. Silencing of PGC-1α in cancer cells suspended their invasive potential and abated metastasis [14]. The mRNA as well as protein level of PGC1α was suppressed in the presence of grifolin. We further demonstrated that PGC1α induced MMP2 and CD44 expression and overexpression of PGC1α restored aggressive phenotype of tumor cells suppressed by grifolin. These results imply that grifolin may hamper ROS generation, successive PGC1α signaling and ATP depletion to suppress tumor cell adhesion/migration. We previous identified that grifolin acts as ERK1/2 kinase inhibitor. Activation of mitogen- and stress-activated kinase (MSK-1), downstream effector of ERK1/2, was a key event in the signaling cascade to promote PGC1α expression in Huntington's disease [38]. To determine whether ERK1/2 signaling involved in the ROS scavenger and successive effect by grifolin, we examined the intracellular ROS level in the presence of PD98059. In MGC803 cells, ROS level was markedly abated in PD98059 treated group compared to the control; while in 5-8F cells, there was no significant difference. Similar modulations were observed in PGC1α mRNA detection. This may be in that whether ERK1/2 pathway mediating the anti-oxidation effect of grifolin depends on various cell settings.

A critical aspect of the PGC1 co-activators is that they are highly versatile and have the ability to interact with many different TFs. In doing so, they activate distinct biological programs in different tissues [34]. To further identify the specific TFs interacting with PGC1α to activate MMP2 and CD44 transcription, we conducted a Co-IP pulldown assay using anti- PGC1α antibody followed by LC/MS/MS. Combined with TF binding-site prediction and PPI prediction analysis, we presumed that PGC1α may co-activate with TFs: AP-1 (Fra-1/c-Jun) and LSF. LSF fosters a highly aggressive and metastatic phenotype in different hepatocellular carcinoma (HCC) cells and can target fibronectin 1 (FN1) and tight junction protein 1 (TJP1) to mediate HCC metastasis [39]. Fra-1 and c-Jun are among the major AP-1 members which regulate MMPs expressions. Fra-1 reportedly regulates the high expression of MMP-1,2,9 in nasopharyngeal carcinoma [40]. AP-1 (Fra-1/c-Jun) mediated induction of MMP-1,2 were required for invasion of osteosarcoma cells and angiogenesis of human dermal microvascular endothelial cells (HDMVECs) [41, 42]. We confirmed that PGC1α bound to Fra-1 and LSF, but not c-Jun; and the PPIs were attenuated in the presence of grifolin. Collectively, PGC1α may promote MMP2 and CD44 expression as a co-activator of metastatic-associated TFs: Fra-1 and LSF. And the PPIs were suppressed in the presence of grifolin.

Overall, grifolin may potentially develop as a promising lead compound in the intervention of tumor aggressive progression through targeting the interplay between PGC1α and AP-1/LSF-MMP2/CD44 signaling.

## MATERIALS AND METHODS

### Cell culture

The human nasopharyngeal carcinoma 5–8F and 6-10B [43, 44], human gastric MGC803 cell lines were grown in RPMI 1640 media. The human breast MDA-MB-231(ATCC HTB-26) cancer cell line was grown in DMEM. All were supplemented with 10% v/v heat-inactivated foetal bovine serum (FBS), 1% w/v glutamine and 1% w/v antibiotics and cultured at 37°C in a humidified incubator containing 5% CO2. MGC803 cells were obtained from Beijing institute for cancer research.

### Reagents and chemicals

The antibody against PGC1α and Fra-1 were obtained from Santa Cruz Biotechnology. The antibody against MMP2 was from R&D Systems. The antibody against CD44 and c-Jun were purchased from Cell Signaling Technology. Anti-LSF antibody was from Millipore. Alexa-Fluor-594-conjugated anti-rabbit-IgG and Alexa-Fluor-488- conjugated anti-mouse-IgG antibodies were obtained from Invitrogen. SensoLyte® 520 MMP-2 Assay Kit was purchased from ANASPEC. PD98059 was obtained from Calbiochem and MMP Inhibitor GM6001 from Millipore. The coding region of human PGC1α was cloned by PCR and inserted into GV287 vector by AgeI and BamHI to construct the eukaryotic expression PGC1α plasmid. PGC1α siRNA: 5′-GAGCAAGTATGACTCTCTG-3′, 5′-CAGAGAGTC ATACTTGCTC-3′.

Grifolin (2-trans, trans-farnesyl-5-methylresorcinol) was provided by Kunming Institute of Botany, the Chinese Academy of Sciences (purity > 99%, HPLC analysis). Dimethyl sulphoxide (DMSO, Sigma) was used to dissolve grifolin. The final concentration of DMSO in the culture media was kept less than 0.1% v/v which had no significant effect on the cell growth.

### Wound-healing migration assay

Scratch wound-healing assays were performed in 24-well tissue culture plates. The cells were seeded at a density of 1X10^5^ cells/well. Scratches were made using 1 mL sterile pipette tips and the wells were washed twice with medium. Cells were allowed to grow for additional 24 h or 48 h in the presence or absence of grifolin. Images were taken under a Leica DMI3000 inverted microscope. Gap distance of the wound (μm) was measured using LAS V3.8 software. The alteration of cell migrated distance was calculated using the formula of (B-A), Where, A denotes the gap distance at 24 h or 48 h, and B for gap width at 0 h.

### Cell adhesion assay

For cell-substratum adhesion assays, 96-well tissue culture plates were coated with 0.5mg/mL matrigel in PBS at 37°C for 1 h, and then covered with serum-free DMEM containing 2% BSA for another 1 h. After being treated with 0, 40μM grifolin for 24 h, cells were detached with 10 mmol/L EDTA in DMEM, washed twice with DMEM, and plated in quadruplicate onto the matrigel-coated wells at 2.5×10^5^ cells per well. Cells in three wells of the quadruplicate were allowed to adhere to the matrigel-coated surface for 40 min, followed by four intensive washes with DMEM to remove nonadherent cells, and then incubated in 5μg/mL 3-(4,5-dimethylthiazol-2-yl)-5-(3-carboxymethoxyphenyl)-2-(4-sulfophenyl)-2H- tetrazolium(MTS; Promega) in complete medium at 37°C for 1 h. One of the quadruplicate wells was used for a cell number standard. The absorbance was measured on a Biotek ELx800 spectrophotometer at 490 nm. Values for the triplicate wells were divided by the corresponding cell number standard value to yield relative A_490_, which were subsequently normalized to the average of the control for comparison purposes.

### Cellular ATP measurement

Intracellular ATP concentration was measured by using an ATP bioluminescent somatic cell assay kit (Perkin Elmer). Cells were plated in white 96 well tissue culture microplates at 2×10^4^ cells/well and incubated in 37 °C/5% CO2 incubator for 24 h. The cells were exposed to 0, 40μM grifolin or 40μM PD98059 for additional 24 h. Luminescent intensity from each well was measured using a EnSpireTM 2300 Multilabel reader (Perkin Elmer).

### Measurement of intracellular ROS

Fluorescence image analysis was used to determine the relative levels of ROS. Cells were harvested and suspended at 1×10^6^ cells/ml in PBS. The relative levels of intracellular ROS were analyzed using the cell-permeable superoxide-sensitive fluorochrome 2′,7′-dichlorofluorescein (Invitrogen). Cells were incubated with CM-H_2_DCFDA (2μM) for 15 minutes at 37 °C before analysis using a BD FACSCanto™ II flow cytometry (BD Biosciences). Mitochondrial ROS level was determined by using a specific mitochondrial H_2_O_2_ probe, MitoPY1 (Sigma). Cells were incubated with 10μM MitoPY1 and 100nM MitoTracker(a mitochondrion-selective probe, Invitrogen) for 30 min at 37 °C, then washed and analyzed by TCS SP8 confocal laser microscope (Leica). The fluorescent intensity of MitoPY1 was quantified using the NIH ImageJ.

### Immunofluorescence staining

The cells were grown on glass coverslips overnight, treated with DMSO or grifolin for 24 h, fixed with 4% paraformaldehyde, washed with phosphate-buffered saline (PBS) for 10 min and then permeabilized using 0.3% Triton X-100 in PBS. After permeabilization, the cells were blocked with 5% BSA for 1 h and then incubated with first antibody overnight. The coverslips were washed with PBS and then incubated with Alexa-Fluor-594- conjugated anti-rabbit-IgG or Alexa-Fluor-488- conjugated anti-mouse-IgG secondary antibody for 1 h. The coverslips were then washed and mounted using DAPI, and images were obtained using TCS SP8 confocal laser microscope (Leica).

### Western blotting

Cells were harvested and washed twice with ice-cold PBS, and then lysed in whole-cell extract buffer (25 mM Tris–HCl, pH 7.4, 150mM NaCl, 1%NP40, 1 mM EDTA, 5% v/v glycerol). Equal amounts of the total proteins from cell preparations and PageRuler™ molecular weight markers (Fermentas life sciences) were resolved by SDS–polyacrylamide gel electrophoresis and electrotransferred to a PVDF membrane. The membranes were blocked and then incubated with specific primary antibodies according to the manufacturer's recommendations. The primary antibody complexes were then stained with horseradish peroxidase conjugated secondary antibody and developed with the enhanced chemiluminescence detection kit (ECL; Pierce).

### RNA extraction and quantitative RT-PCR

RNAs were extracted using the TRIzol reagent following the protocol established by the manufacturer. Reverse transcriptional PCR was performed using the RevertAid First Strand cDNA Synthesis kit. The qPCR analysis was performed in a 7500 Real Time PCR System (Applied BioSystems) using the SYBR Green Real-Time PCR kit. The PCR reaction conditions were 10 s at 95 °C followed by 40 cycles of 15 s at 95 °C and 60 s at 60 °C. The nucleotide sequences of the primers used are indicated in Supplementary Table S1.

### Statistical analysis

All statistical calculations were performed with the statistical software program SPSS ver.16.0. Differences between various groups were evaluated by a two-tailed Student's t test and a *p* value < 0.05 was considered statistically significant.

## SUPPLEMENTARY MATERIALS AND METHODS




