# Supplementary material for: Grifolin inhibits tumor cells adhesion and migration via suppressing interplay between PGC1α and Fra-1/LSF-MMP2/CD44 axes
Source: Oncotarget. 2016 Sep 10;7(42):68708–20. doi: 10.18632/oncotarget.11929 (PMC5356584; doi:10.18632/oncotarget.11929)
Supplement: Supplementary file 1 [file oncotarget-07-68708-s001.pdf]

Grifolin inhibits tumor cells adhesion and migration via suppressing interplay between PGC1α and Fra-1 / LSF- MMP2 / CD44 axes

SUPPLEMENTARY MATERIALS AND METHODS

One-dimensional SDS-PAGE Fractionation, In-gel Digestion, and Nano–liquid Chromatography–electrospray Ionization LTQ-Orbitrap MS/MS Analysis

Identification of PGC1α interactome in human nasopharyngeal carcinoma CNE2 cells was performed according to the protocol previous described [1].

REFERENCE

1. Steunou AL, Ducoux-Petit M, Lazar I, Monsarrat B, Erard M, Muller C, Clottes E, Burlet-Schiltz O, Nieto L. Identification of the hypoxia-inducible factor 2α nuclear interactome in melanoma cells reveals master proteins involved in melanoma development. Mol Cell Proteomics. 2013; 12:736-48.

SUPPLEMENTARY FIGURES AND TABLES

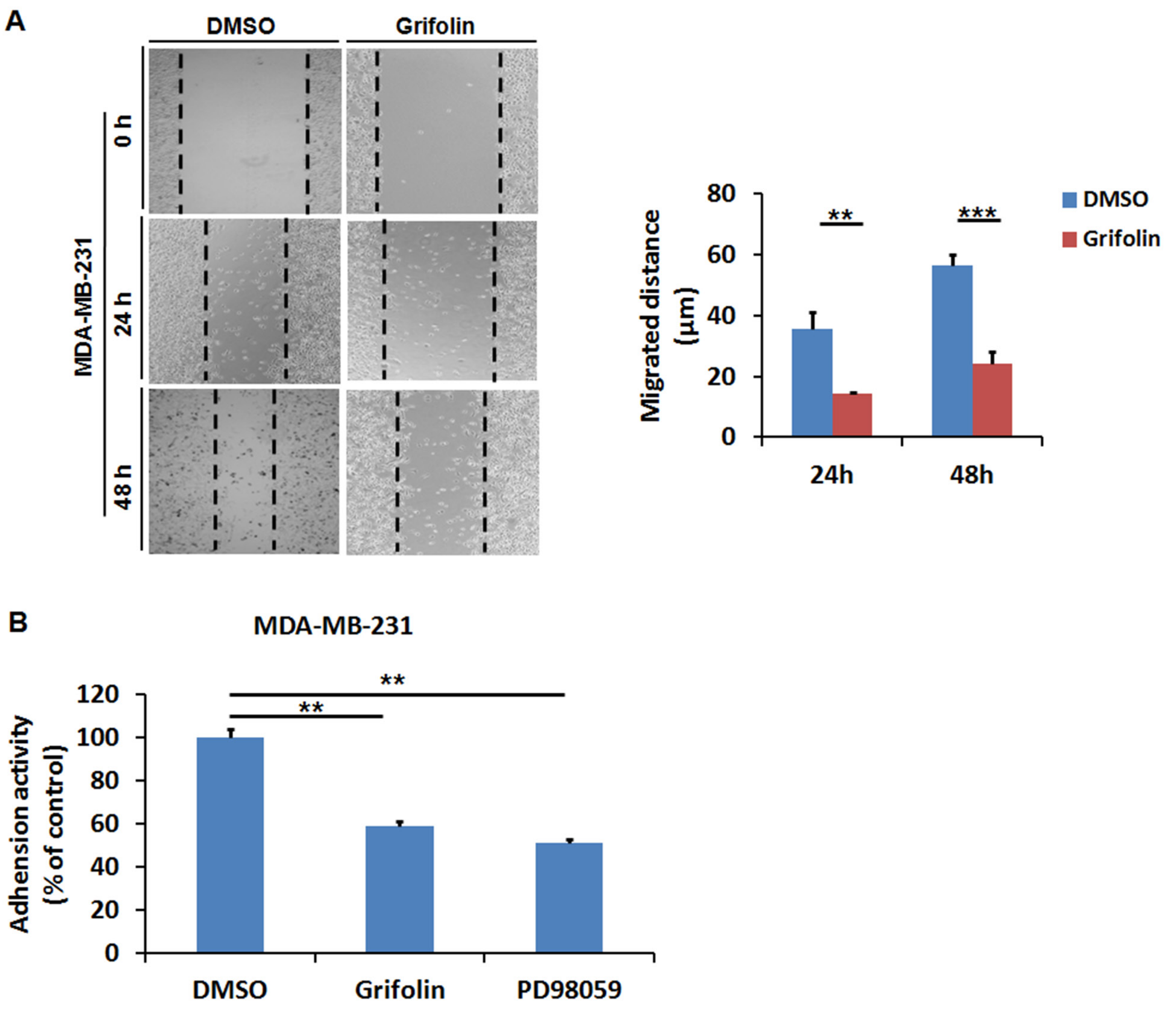

Supplementary Figure S1: Grifolin inhibits migration and adhesion in high-metastatic MDA-MB-231 cells. A. Grifolin suppresses migration in MDA-MB-231 cells using wound healing assay. **B.** Grifolin decreases adhesion in MDA-MB-231 cells. Data are shown as mean values ± S.D. of independent, triplicate experiments. The asterisks (**,***) indicate a significant difference ($p < 0.01$, $p<0.001$) compared to the DMSO control.

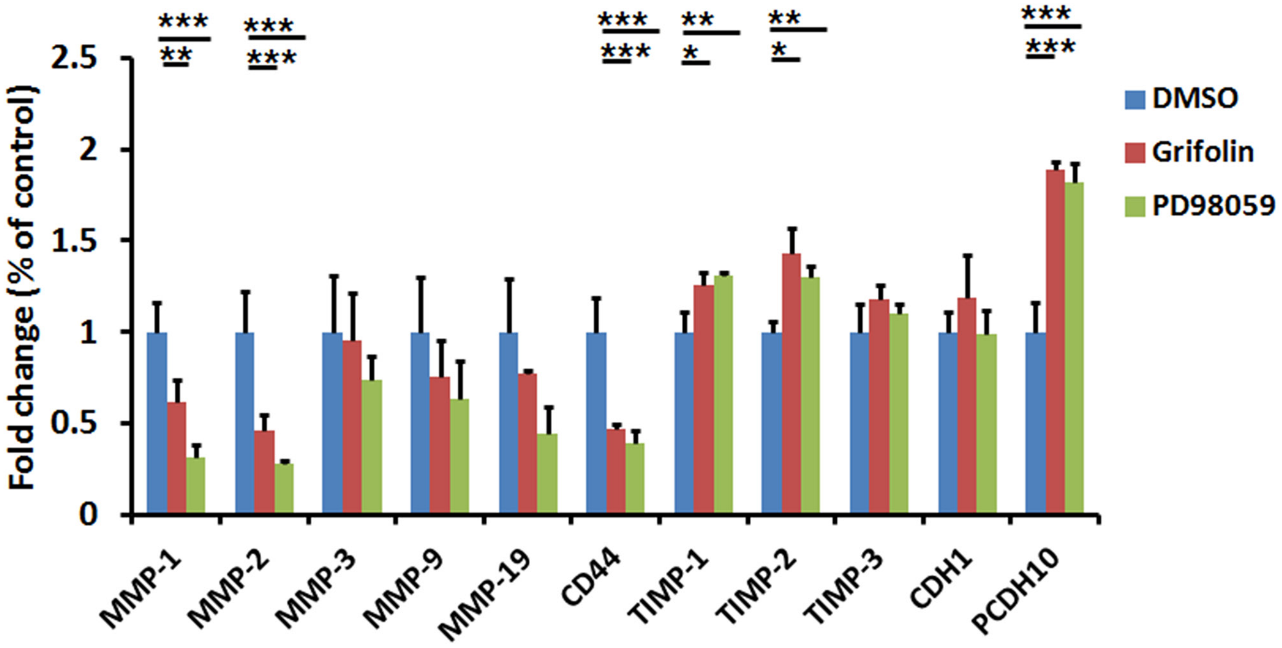

Supplementary Figure S2: A screening of genes related to tumor adhesion/invasion regulation after grifolin treatment. 5-8F cells were incubated with DMSO or 40μM grifolin for 24 hours. The mRNA expressions of genes related to tumor invasion/metastasis were examined by real-time PCR, and GAPDH served as the normalization gene. Data are shown as mean values ± S.D. of independent, triplicate experiments. The asterisks (*,**,***) indicate a significant difference ($p < 0.05$, $p < 0.01$, $p < 0.001$, respectively) compared to the DMSO control.

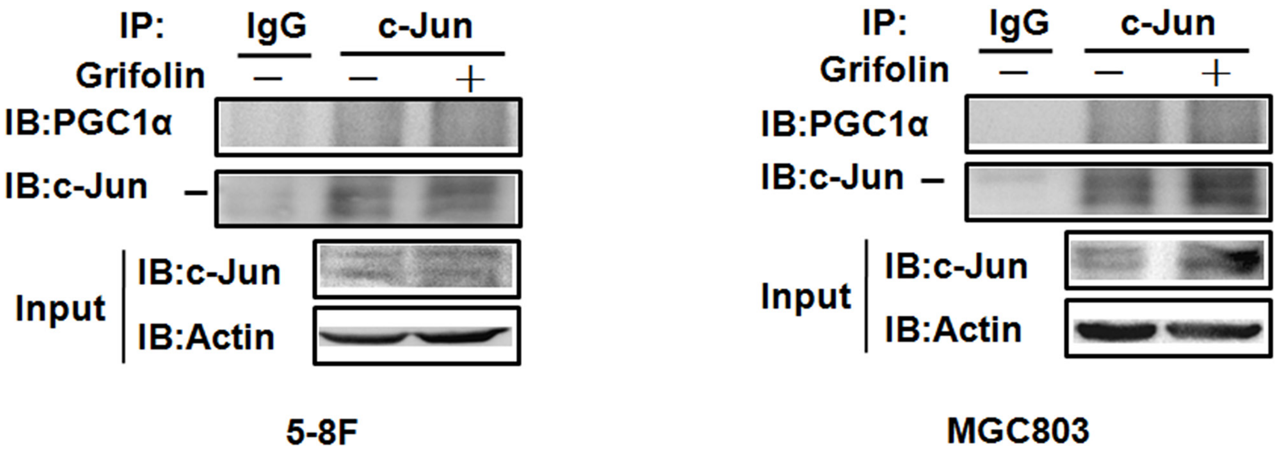

Supplementary Figure S3: Protein-protein interaction interfered by grifolin treatment. 5-8F and MGC803 cells were treated with grifolin (40μM) for 24 h and cell lysates were immunoprecipitated with anti-c-Jun antibody and analyzed by western blot for PGC1α. Immunoprecipitation using non-immune IgG was used in parallel as a control.

Supplementary Table S1: The nucleotide sequences of the primers related to RNA extraction and quantitative RT-PCR section

Target gene		Primer's sequence 5' - 3'
MMP1	Forward	CTTGCACTGAGAAAGAAGACAAAGG
	Reverse	ACACCCCAGAACAGCAGC A
MMP2	Forward	CTCCCGGAAAAGATTGATG
	Reverse	GGTGCTGGCTGAGTAGAT
MMP3	Forward	GAGGAGCTAGCAGGTTATCCTAA
	Reverse	AGCTACACAGTGCTTCTGAACAT C
MMP9	Forward	GCAGATTCCAAACCTTTGAG
	Reverse	GCAAGTCTTCCGAGTAG T
MMP19	Forward	GGGTCCTGTTCTTCCTACAT
	Reverse	CAATCCTGCAGTACTGGTCT
CD44	Forward	TGCCGCTTTGCAGGTGTAT
	Reverse	GGCCTCCGTCCGAGAGA
TIMP1	Forward	CTGCGGATACTTCCACAGGTC
	Reverse	GCAAGAGTCCATCCTGCAGTT
TIMP2	Forward	ATAAGCAGGCCTCCAACGC
	Reverse	GAGCTGGACCAGTCGAAACC
TIMP3	Forward	GC AGATAGACTCAAGGTGTGTGAAA
	Reverse	TCCCTCACTCTTACATGCAGACA
CDH1	Forward	GGCGCCACCTGGAGAGA
	Reverse	TGTCGACCGGTGCAATCTT
PCDH10	Forward	AGTA CGGACACTGAGCACAACC
	Reverse	CGGCGAGGTCTGTCAACTAGATAG
PGC1α	Forward	TGAAGACGGATTGCCCTCATT
	Reverse	GCTGGTGCCAGTAAGAGCTT
GAPDH	Forward	TGTTGCCATCAATGACCCCTT
	Reverse	CTCCACGACGTACTCAGCG

Supplementary Table S2: Identification of PGC1α interactome in human nasopharyngeal carcinoma cells.

See Supplementary File 1
